# A nomogram to predict the probability of axillary lymph node metastasis in early breast cancer patients with positive axillary ultrasound

**DOI:** 10.1038/srep21196

**Published:** 2016-02-15

**Authors:** Si-Qi Qiu, Huan-Cheng Zeng, Fan Zhang, Cong Chen, Wen-He Huang, Rick G. Pleijhuis, Jun-Dong Wu, Gooitzen M. van Dam, Guo-Jun Zhang

**Affiliations:** 1 The Breast Center, Cancer Hospital of Shantou University Medical College, Guangdong, China; 2 Department of Medical Oncology, University of Groningen, University Medical Center Groningen, the Netherlands; 3 Guangdong Provincial Key Laboratory for Breast Cancer Diagnosis and Treatment, Cancer Hospital of Shantou University Medical College, Guangdong, China; 4 Department of Ultrasound Diagnosis, Cancer Hospital of Shantou University Medical College, Guangdong, China; 5 Department of Internal Medicine, Medical Spectrum Twente, Enschede, The Netherlands; 6 Department of Surgery, Nuclear Medicine and Molecular Imaging, University of Groningen, University Medical Center Groningen, the Netherlands; 7 Cancer Research Center, Shantou University Medical College, Guangdong, China

## Abstract

Among patients with a preoperative positive axillary ultrasound, around 40% of them are pathologically proved to be free from axillary lymph node (ALN) metastasis. We aimed to develop and validate a model to predict the probability of ALN metastasis as a preoperative tool to support clinical decision-making. Clinicopathological features of 322 early breast cancer patients with positive axillary ultrasound findings were analyzed. Multivariate logistic regression analysis was performed to identify independent predictors of ALN metastasis. A model was created from the logistic regression analysis, comprising lymph node transverse diameter, cortex thickness, hilum status, clinical tumour size, histological grade and estrogen receptor, and it was subsequently validated in another 234 patients. Coefficient of determination (R^2^) and the area under the ROC curve (AUC) were calculated to be 0.9375 and 0.864, showing good calibration and discrimination of the model, respectively. The false-negative rates of the model were 0% and 5.3% for the predicted probability cut-off points of 7.1% and 13.8%, respectively. This means that omission of axillary surgery may be safe for patients with a predictive probability of less than 13.8%. After further validation in clinical practice, this model may support increasingly limited surgical approaches to the axilla in breast cancer.

Axillary lymph node (ALN) status is one of the most important prognostic factors in patients with primary breast cancer and provides critical information for making treatment decisions^1^,^2^. Axillary lymph node dissection (ALND) is a standard surgical approach for all patients in the 20^th^ century to both assess ALN status and treat metastatic ALNs. However, as many as about 70% of early breast cancer patients exhibit no ALN metastasis. In these cases, ALND can be deemed as a significant overtreatment with its accompanying morbidity such as pain, shoulder range of motion impairment, arm lymphedema, and paresthesia or numbness^3^,^4^,^5^,^6^.

During the past 15 years, sentinel lymph node biopsy (SLNB) has been widely used as an alternative to ALND in clinically node negative patients. SLNB has improved the management of clinically ALN negative patients and reduced significantly morbidity. However, SLNB is an expensive and time-consuming procedure due to the need for pathological assessment of the SLN during the operation. Because of the significant risk of morbidity mentioned above and the disadvantages of SLNB, several randomized clinical trials were performed to assess if avoiding axillary surgery is safe in patients with low risk of ALN metastasis. The results from these studies showed similar disease free survival and overall survival in patients with ALND or without any axillary surgery^7^,^8^. Nowadays, indications for the management of ALND become much stricter due to the promising results from several randomized controlled clinical trials, such as the AMAROS trial^9^,^10^,^11^. These trials demonstrated that either axillary radiotherapy^9^ or omission of further axillary surgery^10^,^11^ could be a safe alternative to ALND in highly selected patients with limited sentinel lymph node (SLN) metastasis, without compromising overall survival, nor regional disease control, although one study showed that an estimated 11–27% of patients have residual nodal disease which was not removed by surgery^10^.

Based on the above findings, there remains considerable doubt about the value of the SLNB in selected patients. A randomized trial has been launched in Italy to evaluate whether the SLNB can be safely omitted in patients with clinically negative ALN^12^. The result of the trial is still to be expected. Seventy-five percent of patients have pathologically negative axillary nodes in those with preoperative negative ultrasound^13^. Meanwhile, among patients with a preoperative positive ultrasound, around 40% have negative axillary nodes upon final pathological examination of ALND specimen^14^. Therefore, it is of great clinical relevance to select those patients that can safely omit axillary surgery or should not undergo either a SLNB or an ALND.

Clearly, there is a need for the development of a decision-making tool in order to reliably select patients unlikely having ALN involvement. The aim of this study is to develop and validate a model to predict the probability of ALN metastasis in early breast cancer patients with positive axillary ultrasound, providing a preoperative tool for assisting clinical decision-making. We generated the model with a training cohort of 322 patients and validated it in an additional set of 234 patients from the same institution. A nomogram was created as a graphical representation of the model. In addition, a user-friendly web-based calculator was developed to facilitate use of the model in a clinical setting while providing complete insight in the model’s parameters.

## Results

### Clinicopathological characteristics of patients

The descriptive clinicopathological characteristics of patients in the modeling and validation group are provided in Table 1. No statistically significant differences were found between the two groups, except for P53, estrogen receptor (ER), and ALN variables including number of lymph nodes detected by ultrasound, transverse diameter, cortical thickness and absence of hilum.

197 patients (61.2%) positively expressed P53 in the modeling group, compared with 191 patients (81.6%) in the validation group. With regard to ER, the positive expression rate between the modeling group and validation group were quite similar, being 63% and 66.7%, respectively. However, the distribution of ER positive patients in the two groups were quite different, with a larger percentage of patients being ER 2+ in the modeling cohort (17.7%) compared to the validation cohort (9.0%), and with relatively smaller percentage in ER 1+ (6.8% versus 10.7%) and ER 3+ (38.5% versus 47.0%) in the modelling group. We considered that the difference might due to the relatively small number of patient population.

**Figure 1:**
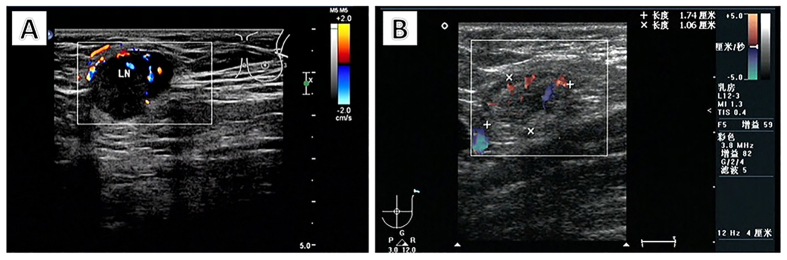
Ultrasound findings of positive and negative axillary lymph nodes. A positive lymph node (**A**) was detected in the left axilla with thickened cortex, and the hilum is disappeared. The diameters are 19 × 16 mm. A negative lymph node (**B**) was detected in the left axilla with normal cortex and hilum. The diameters of the lymph node are 21 × 6 mm.

The absolute median number of transverse diameter and cortical thickness between the modeling group and the validation group matched closely, with 13 mm versus 15 mm, and both 4 mm, respectively. A total of 54.7% of patients lost their lymph node hilum in the validation group, compared with only 39.1% in the modeling group. More than one lymph node was found in 199 patients (61.8%) in the modeling group, compared with 166 patients (70.9%) in the validation group. Typical ultrasound findings of positive and negative lymph nodes are shown in Fig. 1.

Lymph node metastasis was detected in 163 patients (50.6%) in the modeling group, compared with 133 patients (56.8%) in the validation group. Results of comparison of lymph node metastasis by clinicopathological variables in the modeling group are shown in Table 2.

### Univariate and multivariate analyses in the modeling group

In the univariate analysis, factors that were significantly associated with ALN metastasis included clinicopathological variables of primary tumour including clinical tumour size (p = 0.037), histological grade (p < 0.001), ER (p = 0.002), progesterone receptor (PR) (p = 0.001), molecular subtype (p = 0.009), and lymph node variables assessed by ultrasonography including number of nodes (p = 0.010), transverse diameter (p < 0.001) and longitudinal diameter of lymph node (p < 0.001), longitudinal-to-transverse ratio (p = 0.001), cortical thickness (p < 0.001), absence of medulla (p < 0.001), and absence of hilum (p < 0.001) (Table 3).

In the multivariate logistic regression analysis, transverse diameter (p = 0.044), cortical thickness (p = 0.002), absence of hilum (p = 0.001), clinical tumour size (p = 0.018), histological grade (p < 0.001), and ER (p = 0.001) were identified as independent predictors of ALN metastasis and were included in the predictive model. Histological grade III showcases the highest predictive value with an OR of 8.083, in comparison with grade I tumours (95% CI, 3.022-21.616) (Table 3).

### Predictive equation, nomogram and web-based calculator

An equation, ln (p/1 − p) = 0.063 × a + 0.277 × b + 1.420 × c + 1.502 × d1 + 2.090 × d2 + 0.305 × e + 0.379 × f − 5.710, was generated to evaluate the probability of ALN metastasis preoperatively based on the result from the multivariate logistic regression analysis in the modeling group.

The denotement of letters in the equation is as follows: p = the probability of ALN metastasis; a = transverse diameter of lymph node as detected by ultrasound in mm; b = cortical thickness of lymph node as detected by ultrasound in mm; c = hilum (0 if present, 1 if absent); d1 = histological grade 1 (0 if grade 1 (G1) or grade 3 (G3), 1 if grade (G2)); d2 = histological grade 2 (0 if G1 or G2, 1 if G3); e = clinical tumour size in cm; f = ER (0 if−, 1 if +, 2 if ++, 3 if +++).

**Figure 2:**
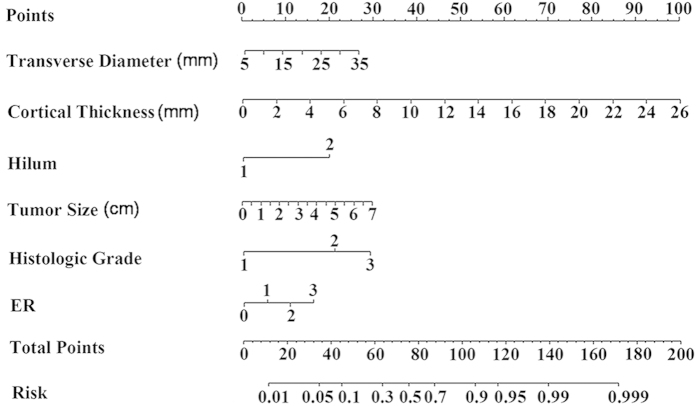
Nomogram for predicting the probability of axillary lymph node metastasis. The nomogram comprises nine rows. The first row is the point assignment for each variable. For an individual patient, each variable is assigned a point value according to the clinicopathological characteristics by drawing a vertical line between the exact variable value and the Points line. Subsequently, a total point (row 8) can be obtained by summing all of the assigned points for the six variables. Finally, the predictive probability of axillary metastasis can be obtained by drawing a vertical line between Total Points and Risk (the final row).

A nomogram was developed based on the result of multivariate logistic regression analysis (Fig. 2). The nomogram comprises nine rows and representation of each row is as follows. The first row (Points) is the point assignment for each variable. For an individual patient, each variable is assigned a point value according to the clinicopathological characteristics by drawing a vertical line between the exact variable value and the Points line. Subsequently, a total point score (row 8) can be calculated by summing all of the assigned points for the six variables. The predictive probability of axillary metastasis can be obtained by drawing a vertical line between Total Points and Risk (the final row). In addition, a web-based calculator was developed to facilitate use of the model in a clinical setting. The web-based calculator is freely accessible at https://app.evidencio.com/models/show/170.

**Figure 3:**
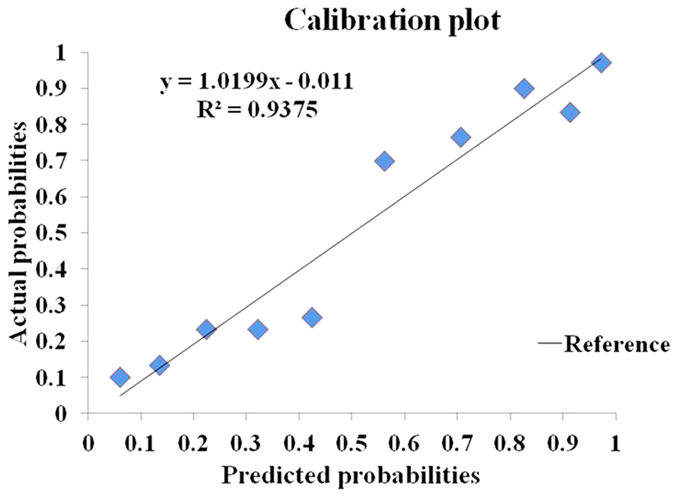
Calibration of the predictive model in the modeling group. All patients were grouped into deciles according to their predicted probabilities. The mean predicted probability of each decile was plotted against the actual probability of axillary lymph node (ALN) metastasis. The reference line represents perfect equality of the predicted probability and the actual incidence of ALN metastasis. The coefficient of determination (R^2^) reflects the calibration of the model. The calibration of the model was considered acceptable with R^2^ of 0.9375.

**Figure 4:**
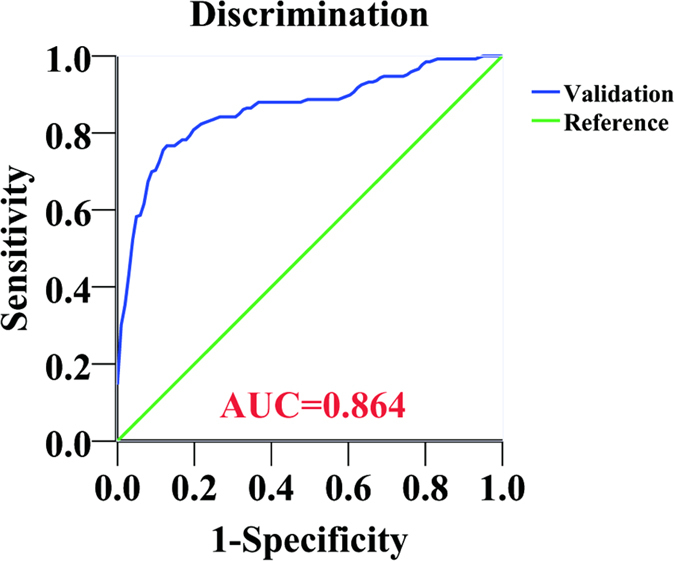
Receiver-operating characteristic (ROC) curve of the predictive model for the validation group. The reference line with a slope of 1 indicates an area under the ROC curve value of 0.5, which means that the probability of axillary lymph node (ALN) metastasis is equal to the toss of a coin. An area under the ROC curve (AUC) value of 1 represents perfect discrimination of a patient with positive ALN from the one with negative ALN. The model showed good performance with AUC of 0.864.

The mean predicted probability and mean actual rate of axillary metastasis in each decile were calculated. Calibration of the model was considered acceptable with R^2^ of 0.9375 (Fig. 3). The Hosmer-Lemeshow Goodness-of-Fit test resulted in a p value of 0.18, indicating that the model fits well. The model was then validated with an additional set of 234 patients from the same institution. The performance of the model was good with an AUC of 0.864 (Fig. 4).

Sensitivity, specificity, accuracy and false-negative rate (FNR) of the model for several predicted probability cut-off values in the validation group are shown in Table 4. The FNRs of the model were 0% and 5.3% for the predicted probability cut-off points of 7.1% and 13.8%, respectively.

## Discussion

A precise noninvasive evaluation of ALN status preoperatively, although challenging, is very important for optimization of the treatment plan for patients with early breast cancer. Efforts have been made to construct a more precise and reliable tool for assessing the ALN status by using one, (e.g. ultrasound or digital mammography (MMG)), or a combination (e.g. magnetic resonance imaging (MRI), ultrasound and MMG) of current imaging modalities used in breast cancer patients, but the accuracy kept varying with less satisfactory. The FNRs of axillary ultrasound examination for determining lymph node metastasis were reported as 16.7% and 22.9%^15^,^16^. When combined with other modalities, such as MRI, physical examination, MMG, or positron emission tomography/computed tomography (PET/CT), the FNRs declined slightly, but were still as high as 14–16.9%^15^,^16^. Besides, the MRI and PET/CT examinations are too expensive to be routinely implemented in all patients. Diepstraten *et al.*^13^ reported that the FNRs of ultrasound-guided ALN biopsy ranged from 15% to 40%, with a pooled estimate of 25%, which was much higher than the overall FNR of SLNB reported by the American Society of Clinical Oncology (ASCO)^17^. Moreover, the ultrasound-guided lymph node biopsy is an invasive procedure, which may increase risks like blood vessel damage. Development of a noninvasive, more practical, reliable and accurate clinical decision-making tool for predicting the probability of ALN metastasis is of great importance in clinical practice.

In the present study, we developed a predictive model to estimate the probability of ALN metastasis in early breast cancer patients with positive axillary ultrasound findings. The model was well calibrated in the modeling group and showed good performance for evaluation of nodal metastasis in the validation group with an AUC of 0.864. Some clinicopathological variables showed statistically significant differences between the modeling group and the validation group, which might be due to the relatively small sample size. Despite significant difference existed, the absolute median number of transverse diameter and cortical thickness between two groups matched closely. That explains why the model still performed well in the validation group, although transverse diameter and cortical thickness served as independent risk factors for node metastasis. Our result emphasizes the stability of the model when applied to a different set of patients. Since stability serves as one of the most important quality parameters for a well-developed model, the stability of our model showcased in this study provides us confidence that the model is generally applicable to other Asian patient populations, although further validation in independent patient cohorts is desirable.

According to NSABP B-32^18^, micrometastastic disease was an independent prognostic factor for patients with initially negative SLN. Therefore, in our study, IHC staining was routinely performed to detect micrometastasis when no tumour cells were identified on H&E staining. In a multivariate analysis, six variables emerged as independent predictors for ALN metastasis and were included in the final model. They were clinical tumour size, histological grade, ER, transverse diameter, cortical thickness and absence of hilum of the lymph node as detected by ultrasound. Tumour size and histological grade have been reported to be risk factors for ALN metastasis in many other studies^19^,^20^,^21^,^22^,^23^,^24^, as could be confirmed by our results. The predictive value of ER and PR status in previous studies was uncertain, with some studies showing no predictive value for ER and PR status^19^,^21^,^22^,^24^, and others reporting that lower risk of ALN metastasis was found in tumours with negative expression of either ER^25^ or PR^23^,^26^. In this study, we found that ER overexpression was associated with higher probability of ALN metastasis. This finding may seem counterintuitive, but is similar to the findings from Bevilacqua *et al.*^20^. Although we do not know the actual reason of this phenomenon, we hypothesize that ER negative tumours may prefer hematogenous metastasis rather than lymphatic metastasis.

To the best of our knowledge, this is the first ALN metastasis predictive model mainly incorporating ultrasound parameters reported in the English literature. Preoperative axillary ultrasound is a simple test that is routinely used for assessing the clinical ALN status in breast cancer patients. According to previous reports, thickening of cortex and disappearance of hilum were associated with lymph node metastasis^14^,^16^. The result of our study is in line with those findings. In our final nomogram, transverse diameter, cortical thickness and hilum status were included as independent predictors of ALN metastasis. For an expert radiologist, axillary ultrasound can almost always identify lymph nodes and access the node morphology^27^,^28^,^29^. In that case, the three ultrasound parameters incorporated in our model could be easily obtained in almost all breast cancer patients, which will ensure the application of the model. Around 15–40% of patients with positive axillary ultrasound may have tumour-negative nodes on pathological evaluation^14^,^30^,^31^,^32^. Obviously, it is of great value to identify those patients preoperatively, for whom SLNB or even omitting surgical intervention may be a suitable option. We created the predictive model based on data from patients with positive axillary ultrasound findings in order to select those patients with a low risk of node metastasis.

In our study, patients with a predictive probability of less than 7.1% presented with no apparent lymph node metastasis. When the predicted probability cut-off point was set at 13.8%, the FNR of the model was only 5.3%, much lower than the FNR of SLNB reported by ASCO (overall 8.4%, range 0–29%)^17^. In that condition, omission of SLNB appears to be acceptable after thorough discussion with patients, especially in elderly patients with comorbid conditions. The pathological ALN status is not the only determinant to decide whether a patient should receive systemic adjuvant therapy or not. Histological grade, molecular subtype, tumour type, tumour size, and age of the patient should also be taken into consideration. In our study, only two patients, with an estimated predictive value of less than 13.8%, did not receive systemic therapy according to local guidelines. That is to say, patients would not be undertreated in terms of systemic therapy although they did not receive axillary surgery. Our model could provide doctors with a tool to estimate the probability of ALN metastasis and help them weigh the risks and benefits of SLNB more appropriately. Ultimately, this may prevent selected patients from undergoing unnecessary axillary surgery and associated comorbidity.

To our knowledge, several models have been developed to predict the probability of ALN metastasis, with reported AUC ranging from 0.702 to 0.79^20^,^21^,^33^,^34^. Among these models, the model developed by Bevilacqua *et al.* from the Memorial Sloan-Kettering Cancer Center (MSKCC) in 2007 is the most widely used. The MSKCC model produced an AUC of 0.754 when validated in another patient cohort from the same center^20^. Subsequently, the model was validated in a German breast cancer patient cohort^24^ and two Chinese breast cancer patient cohorts^25^,^33^, with an AUC of 0.78, 0.71 and 0.73, respectively, all showing a reasonable ability in distinguishing patients with positive lymph nodes from those with negative nodes. The MSKCC model incorporates nine parameters (tumour type, pathological tumour size, lymphovascular invasion (LVI), tumour location, age, multifocality, nuclear grade, ER and PR), some of which are available only post-operatively, such as pathological tumour size, LVI, and multifocality. This may limit the clinical implication of the model, and thus a second surgical procedure might be needed. Two more predictive models reported by Takada *et al.*^34^ and Chen *et al.*^33^ also require parameters that are not available preoperatively. We believe that, when compared with those models, our model shows some advantages. First, our model contains only six variables that are all routinely available prior to surgery, e.g. histological grade and hormone receptor status can be obtained after core needle biopsy of the primary tumour. This is expected to facilitate the clinical utility of the model in different settings, irrespective of infrastructure. Second, our model showed an excellent discrimination, yielding an AUC of 0.864. By calculating the individual probability of ALN metastasis using our user-friendly web-based calculator, doctors could inform their patients regarding their chances of having a positive ALN preoperatively. This knowledge will allow patients to better participate in discussing the treatment strategy on axillary nodes and make an informed decision.

Although our model provides a promising predictive value in early breast cancer patients, it still has some limitations. First, the model was only validated in one small internal validation cohort (n = 234). It needs to be further validated in external validation groups to evaluate its predictive ability and generalizability. Second, risk factors like clinical tumour size, cortical thickness and transverse diameter of lymph node may differ when measured by different doctors due to inter-observer variability. Taking the above-mentioned into account, our model cannot be considered an alternative to SLNB, even in patients with a low predicted probability of ALN metastasis. Validation studies using independent datasets to judge our model on its merits are encouraged. After further validation in clinical practice, this model may support increasingly limited surgical approaches to the axilla in breast cancer.

## Methods

### Study design and patients population

This study enrolled a consecutive series of 322 patients with primary invasive early breast carcinoma treated at the Breast Center, Cancer Hospital of Shantou University Medical College from November 2009 to April 2014 for developing the predictive model. An additional set of 234 consecutive patients treated at the same institution between May 2014 and November 2015 were enrolled as the validation group.

The inclusion criteria were female patients with early invasive breast cancer (clinical TNM stage according to the 7^th^ edition of the American Joint Committee on Cancer (AJCC) Cancer Staging Manual^35^: T1-3 and N0-1), having positive axillary ultrasound findings (defined as at least one lymph node visible by ultrasound^36^), and receiving a successful SLNB or ALND. Patients with local advanced disease (TNM stage according to the 7^th^ edition of the AJCC Cancer Staging Manual^35^: T4 or N2-3), neo-adjuvant treatment, or bilateral breast cancer were excluded.

All patients underwent a preoperative axillary ultrasound assessment using the IU22 (PHILIPS, The Netherlands) and ACUSON S2000 (SIEMENS, Germany) with a high-frequency transducer (12 to 15 MHz). Characteristics of lymph nodes including number of nodes, transverse diameter, longitudinal diameter, longitudinal-to-transverse axis ratio, cortical thickness, absence of medulla and absence of hilum were recorded. The ultrasound findings were reviewed and re-assessed by an experienced radiologist (C.C.) with more than 10 years of experience in breast ultrasound imaging. Surgical treatment for the primary disease included breast-conserving surgery or mastectomy with or without breast reconstruction. Systemic and radiation therapy were performed according to the local clinical practice guideline.

We utilized each of the following variables: age at diagnosis, menopausal status, clinical tumour size, tumour location, histological type, histological grade, ER, PR, human epidermal growth factor receptor 2 (Her-2), Ki-67, P53, vascular endothelial growth factor C (VEGF-C), as well as molecular subtype, and all above-mentioned variables of lymph node on ultrasonography. If more than one lymph node was detected, the variables were assessed on the lymph node most suspicious for metastasis.

Tumour tissues were obtained from paraffin embedded specimens for hematoxylin and eosin (H&E), immunohistochemistry (IHC), and fluorescence *in situ* hybridization (FISH) staining. The technique of histopathological analysis has been described previously^37^. Grading of invasive tumour was scored according to the Nottingham (Elston-Ellis) modification of the Scarf-Bloom-Richardson grading system. All nodes were examined postoperatively with serial section H&E staining. IHC staining was performed to determine whether micrometastasis (0.2–2 mm cancer foci) existed or not when no cancer cells were identified on H&E staining. ER^37^, PR^37^, P53^37^, and VEGF-C^38^ were considered positive if immunostaining was positive in more than 10% of tumour cells. Her-2 positivity was defined as a score of 3+ on IHC^37^ or amplification on FISH^39^. Ki-67 was considered positive if nuclear staining of tumour cells was >14% and negative if ≤14%^19^.

### Statistical analysis

Differences of continuous variables between groups were analyzed using the Mann-Whitney U test. The chi-square test was performed to compare the rates between different groups. In the modeling group, a univariate analysis was performed to assess risk factors for ALN metastasis. Variables with a p-value less than 0.05 in the univariate analysis were included in a binary logistic regression analysis using a backward selection procedure in order to discover the independent risk factors of ALN metastasis. Variables with a p-value less than 0.05 in the multivariate analysis, as an independent risk factor, were included in an equation for predicting the probability of ALN metastasis. Coefficients for each variable and the constant in the equation were generated based on multivariate analysis. A nomogram was developed to be a graphic representation of the model. To calibrate the model, the patients in the modeling group were grouped into deciles with respect to their predicted probabilities of lymph node metastasis calculated by the equation. For each decile, the mean predicted metastatic probability was compared with the mean actual metastatic rate. The calibration was assessed graphically and by calculation of the coefficient of determination (R^2^). The predictive model was then validated with an additional set of 234 Chinese patients in the validation group. The receiver operating characteristic (ROC) curve was drawn, and the area under the curve (AUC) was used to assess the predictive accuracy of the model. Fit of the model was evaluated by the Hosmer-Lemeshow Goodness-of-Fit test. Two tailed p-values of less than 0.05 were considered statistically significant. Statistical analysis was performed by using the statistical software SPSS (version 19, SPSS Inc., Chicago, IL) and “R” (version 3.1.0).

### Ethical approval

This study has been approved by the Ethics Committee of the Cancer Hospital of Shantou University Medical College, and was performed in accordance with the ethical standards laid down in the 1964 declaration of Helsinki and all subsequent revisions. All persons mentioned in the paper gave their informed consent prior to inclusion in the study.

## Additional Information

**How to cite this article**: Qiu, S.-Q. *et al.* A nomogram to predict the probability of axillary lymph node metastasis in early breast cancer patients with positive axillary ultrasound. *Sci. Rep.* **6**, 21196; doi: 10.1038/srep21196 (2016).

## Figures and Tables

**Table 1:** Comparison between modeling group and validation group by clinicopathological characteristics.

Characteristic	Modeling No. (%)	Validation No. (%)	p-value
No. of Patients	322 (100%)	234 (100%)	-
Age at diagnosis (year)			0.218
<=35	25 (7.8%)	12 (5.1%)	
>35	297 (92.2%)	222(94.9%)	
Menopausal status			0.155
Premenopausal	182 (56.5%)	118 (50.4%)	
Postmenopausal	140 (43.5%)	116 (49.6%)	
Clinical Tumor size (mm)			0.594
Median (IQR)	30 (23, 40)	30 (24, 40)	
Clinical tumor size			0.355
T1	74 (23.0%)	44(18.8%)	
T2	223 (69.3%)	169(72.2%)	
T3	22 (6.8%)	21(9.0%)	
Unknown	3 (0.9%)	0(0.0%)	
Tumor location			0.457
UOQ	152 (47.2%)	118(50.4%)	
LQQ	42 (13.0%)	25(10.7%)	
UIQ	51 (15.8%)	43(18.4%)	
LIQ	15 (4.7%)	14(6.0%)	
Central	62 (19.3%)	34(14.5%)	
Histological grade			0.085
I	49 (15.2%)	24(10.2%)	
II	104 (32.3%)	95(40.6%)	
III	154 (47.8%)	113(48.3%)	
Unknown	15 (4.7%)	2(0.9%)	
Histological type			0.49
Ductal	294 (91.3%)	220(94.0%)	
Lobular	10 (3.1%)	5(2.1%)	
Other	18 (5.6%)	9(3.9%)	
ER			0.006
Negative	119 (37.0%)	78(33.3%)	
1+	22 (6.8%)	25(10.7%)	
2+	57 (17.7%)	21(9.0%)	
3+	124 (38.5%)	110(47.0%)	
PR			0.654
Negative	132 (41.0%)	106(45.3%)	
1+	38 (11.8%)	30(12.8%)	
2+	63 (19.6%)	39(16.7%)	
3+	89 (27.6%)	59(25.2%)	
Her-2			0.336
Negative	223 (69.3%)	153(65.4%)	
Positive	99 (30.7%)	81(34.6%)	
Ki-67			0.08
<=14	51 (15.9%)	25(10.7%)	
>14	268 (83.2%)	207(88.5%)	
Unknown	3 (0.9%)	2(0.8%)	
P53			<0.001
Negative	117 (36.3%)	38(16.2%)	
Positive	197 (61.2%)	191(81.6%)	
Unknown	8 (2.5%)	5(2.2%)	
VEGF-C			0.231
Negative	75 (23.3%)	43(18.4%)	
Positive	222 (68.9%)	165(70.5%)	
Unknown	25 (7.8%)	26(11.1%)	
Molecular subtype			0.622
Luminal A	173 (53.7%)	119(50.9%)	
Luminal B	44 (13.7%)	38(16.2%)	
HER-2 enriched	51 (15.8%)	43(18.4%)	
Triple negative	54 (16.8%)	34(14.5%)	
Lymph node detected by ultrasound Number			0.025
1	123 (38.2%)	68(29.1%)	
>=2	199 (61.8%)	166(70.9%)	
Transverse diameter (mm)			0.005
Median (IQR)	13 (10, 17)	15 (11, 18)	
Longitudinal diameter (mm)			0.092
Median (IQR)	7 (5, 9)	7 (5.75, 10)	
Longitudinal to transverse ratio			0.441
Median (IQR)	0.53 (0.44, 0.67)	0.53 (0.42, 0.67)	
Cortical thickness (mm)			0.029
Median (IQR)	4 (3, 6)	4 (3, 7)	
Absence of medulla			0.522
Yes	87 (27.0%)	69(29.5%)	
No	235 (73.0%)	165(70.5%)	
Absence of hilum			<0.001
Yes	126 (39.1%)	128(54.7%)	
No	196 (60.9%)	106(45.3%)	
Lymph node metastases			0.147
Yes	163 (50.6%)	133(56.8%)	
No	159 (49.4%)	101(43.2%)	

UOQ: upper outer quadrant; UIQ: upper inner quadrant; LOQ: lower outer quadrant;

LIQ: lower inner quadrant; IQR: Interquartile range, which is the 25^th^ percentile, 75^th^ percentile.

**Table 2:** Comparison of axillary lymph nodes metastasis by clinicopathological variables.

Characteristic	Axillary lymph node metastasis (n = 163)	No axillary lymph node metastasis (n = 159)	All patients (n = 322)	p-value
Age at diagnosis (year)				0.785
<=35	12 (48.0%)	13 (52.0%)	25	
>35	151 (50.8%)	146 (49.2%)	297	
Menopausal status				0.632
Premenopausal	90 (49.5%)	92 (50.5%)	182	
Postmenopausal	73 (52.1%)	67 (47.9%)	140	
Clinical tumor size (mm)				0.007
Median (IQR)	30 (25, 40)	30 (20, 40)	30 (23, 40)	
Clinical tumor size				<0.001
T1	23 (31.1%)	51 (68.9%)	74	
T2	131 (58.7%)	92 (41.3%)	223	
T3	7 (31.8%)	15 (68.2%)	22	
Unknown	2 (66.7%)	1 (33.3%)	3	
Tumor location				0.626
UOQ	72 (47.4%)	80 (52.6%)	152	
LOQ	21 (50.0%)	21 (50.0%)	42	
UIQ	25 (49.0%)	26 (51.0%)	51	
LIQ	9 (60.0%)	6 (40.0%)	15	
Center	36 (58.1%)	26 (41.9%)	62	
Histological grade				<0.001
I	10 (20.4%)	39 (79.6%)	49	
II	49 (47.1%)	55 (52.9%)	104	
III	101 (65.6%)	53 (34.4%)	154	
Unknown	3 (20.0%)	12 (80.0%)	15	
Histological type				0.318
Ductal	152 (51.7%)	142 (48.3%)	294	
Lobular	5 (50.0%)	5 (50.0%)	10	
Other	6 (33.3%)	12 (66.7%)	18	
ER				0.013
Negative	49 (41.2%)	70 (58.8%)	119	
1+	8 (36.4%)	14 (63.6%)	22	
2+	32 (56.1%)	25 (43.9%)	57	
3+	74 (59.7%)	50 (40.3%)	124	
PR				0.009
Negative	54 (40.9%)	78 (59.1%)	132	
1+	20 (52.6%)	18 (47.4%)	38	
2+	32 (50.8%)	31 (49.2%)	63	
3+	57 (64.0%)	32 (36.0%)	89	
Her-2				0.788
Negative	114 (51.1%)	109 (48.9%)	223	
Positive	49 (49.5%)	50 (50.5%)	99	
Ki-67				0.821
<=14	25 (49.0%)	26 (51.0%)	51	
>14	136 (50.7%)	132 (49.3%)	268	
Unknown	2 (66.7%)	1 (33.3%)	3	
P53				0.954
Negative	59 (50.4%)	58 (49.6%)	117	
Positive	100 (50.8%)	97 (49.2%)	197	
Unknown	4 (50.0%)	4 (50.0%)	8	
VEGF-C				0.215
Negative	34 (45.3%)	41 (54.7%)	75	
Positive	119 (53.6%)	103 (46.4%)	222	
Unknown	10 (40.0%)	15 (60.0%)	25	
Molecular subtype				0.007
Luminal A	98 (56.7%)	75 (43.3%)	173	
Luminal B	26 (59.0%)	18 (41.0%)	44	
HER-2 enriched	17 (33.3%)	34 (66.7%)	51	
Triple negative	22 (40.7%)	32 (59.3%)	54	
Lymph node detected by ultrasound Number				0.010
1	51 (41.5%)	72 (58.5%)	123	
>=2	112 (56.3%)	87 (43.7%)	199	
Transverse diameter (mm)				0.001
Median (IQR)	14 (10, 19)	12 (9, 15)	13 (10, 17)	
Longitudinal diameter (mm)				<0.001
Median (IQR)	8 (6, 10)	6 (5, 7)	7 (5, 9)	
Longitudinal to transverse ratio				0.005
Median (IQR)	0.57 (0.44, 0.71)	0.50 (0.43, 0.60)	0.53 (0.44, 0.67)	
Cortical thickness (mm)				<0.001
Median (IQR)	6 (4, 9)	3 (2, 4)	4 (3, 6)	
Absence of medulla				<0.001
Yes	73 (83.9%)	14 (16.1%)	87	
No	90 (38.3%)	145 (61.7%)	235	
Absence of hilum				<0.001
Yes	102 (81.0%)	24 (19.0%)	126	
No	61 (31.1%)	135 (68.9%)	196	

UOQ, upper outer quadrant; UIQ, upper inner quadrant; LOQ, lower outer quadrant; LIQ, lower inner quadrant;

IQR, Interquartile range, which is the 25^th^ percentile, 75^th^ percentile.

**Table 3:** Univariate and multivariate analysis for factors associated with axillary lymph node metastasis.

Characteristic	Univariate				Multivariate		
	OR	95% CI	P-value	Coefficient	OR	95% CI	p-value
Age at diagnosis (year)							
<=35	1						
>35	1.120	0.495–2.536	0.785				
Menopausal status							
Premenopausal	1						
Postmenopausal	0.898	0.578–1.395	0.632				
Clinical tumor size (mm)	1.210	1.011–1.448	0.037	0.305	1.357	1.053–1.748	0.018
Clinical tumor size			<0.001				
T1	1						
T2	3.157	1.804–5.527	<0.001				
T3	1.035	0.372–2.879	0.948				
Tumor location			0.629				
UOQ	1						
LOQ	0.65	0.358–1.180	0.157				
UIQ	0.722	0.329–1.588	0.418				
LIQ	1.083	0.343–3.420	0.891				
Center	0.694	0.329–1.464	0.338				
Histological grade			<0.001				<0.001
I	1				1		
II	3.475	1.570–7.689	0.002	1.502	4.492	1.644–12.271	0.003
III	7.432	3.441–16.054	<0.001	2.09	8.083	3.022–21.616	<0.001
Histological type			0.300				
Ductal	1						
Lobular	0.935	0.265–3.296	0.916				
Other	0.425	0.144–1.253	0.121				
ER	1.303	1.101–1.541	0.002	0.379	1.461	1.166–1.832	0.001
PR	1.338	1.121–1.597	0.001				
Her-2	0.937	0.584–1.504	0.788				
Ki-67							
<=14	1						
>14	1.072	0.589–1.950	0.821				
P53	0.987	0.823–1.183	0.885				
VEGF-C	1.266	0.981–1.633	0.070				
Molecular subtype			0.009				
Luminal A	1						
Luminal B	1.105	0.565–2.165	0.77				
HER-2 enriched	0.383	0.199–0.737	0.004				
Triple negative	0.526	0.283–0.979	0.043				
Lymph node detected by ultrasound Number							
1	1						
>=2	1.817	1.153–2.865	0.010				
Transverse diameter (mm)	1.08	1.034–1.127	<0.001	0.063	1.065	1.002–1.132	0.044
Longitudinal diameter (mm)	1.351	1.213–1.505	<0.001				
Longitudinal-to-transverse ratio	9.798	2.494–38.491	0.001				
Cortical thickness (mm)	1.653	1.451–1.883	<0.001	0.277	1.319	1.106–1.571	0.002
Absence of medulla							
No	1						
Yes	8.401	4.477–15.765	<0.001				
Absence of hilum							
No	1						
Yes	9.406	5.494–16.104	<0.001	1.42	4.137	1.799–9.514	0.001
Constant				−5.710			

UOQ: upper outer quadrant; UIQ: upper inner quadrant; LOQ: lower outer quadrant; LIQ: lower inner quadrant;

IQR: Interquartile range, which is the 25^th^ percentile, 75^th^ percentile; OR: Odds ratio; CI: Confidence interval.

**Table 4:** Sensitivity, specificity and accuracy of our model in low-risk predictive patients in validation group.

Predicted risk	Patient number and percentage (%)	Number of patients with ALN metastasis	Sensitivity (%)	Specificity (%)	Accuracy (%)	FNR (%)
<7.1%	6 (2.6)	0	100	6	100	0
<13.8%	19 (8.1)	1	99.2	17.8	94.7	5.3
<18.2%	23 (9.8)	2	98.5	20.8	91.3	8.7
<20%	29 (23.1)	5	96.2	23.8	82.8	17.2
<30%	55 (23.5)	14	89.5	40.6	74.5	25.5
<40%	78 (33.3)	16	88	61.4	79.5	20.5

Validation group (n = 234): patients with positive lymph node (n = 133, 56.8%); patients with negative lymph node (n = 101, 43.2%).

ALN: axillary lymph node; FNR: false negative rate.
